# Colorectal Microcarcinoids in Association with Long-Term Exposure to Urinary Content: A Case Report and Review of the Literature

**DOI:** 10.1155/2015/806310

**Published:** 2015-04-02

**Authors:** Grace W. Weyant, Dipti M. Karamchandani, Negar Rassaei

**Affiliations:** Department of Pathology, Penn State Milton S. Hershey Medical Center, 500 University Drive, P.O. Box 850, MC H179, Hershey, PA 17033, USA

## Abstract

Long-term exposure of colonic mucosa to urinary content and its association with increased risk of infection, mechanical and biochemical irritation, and malignancy have been described in the literature. Existing case reports and studies depict the low but distinct risk of malignancy in gastrointestinal segments which come in contact with urinary content as a result of surgical correction of urinary tract abnormalities. However, these reports are largely limited to colonic adenocarcinoma and urothelial cell carcinoma. Late urointestinal carcinoma in patients with ileal incorporation into the urinary tract has also been reported. To the best of our knowledge, however, there is only one case report documenting neuroendocrine (NE) cell hyperplasia in colonic mucosa after long-term cystoplasty. Our case is the first to describe microcarcinoids and diffuse NE hyperplasia occurring in a patient with congenital anorectal anomalies, resulting in long-term exposure of colonic mucosa to fecal stream and urinary content. This case, in conjunction with the reported cases in the literature, raises the distinct possibility of an association between exposure of colonic mucosa to urine and long-term development of malignancy, including NE neoplasms.

## 1. Introduction

Gastrointestinal segments are commonly utilized for urinary tract reconstruction when the creation of a conduit or bladder replacement is indicated [1, 2]. Long-term exposure of colonic mucosa to urinary content is associated with risk of infection, mechanical and biochemical irritation, and malignancy [1, 3]. Rare case reports and studies of malignancy, particularly colonic adenocarcinoma and urothelial cell carcinoma, have been reported in the literature in this setting [1, 4, 5]. Rarely, neuroendocrine (NE) cells, which are usually inconspicuous occupants within the colonic mucosa, may become hyperplastic [2, 6]. We herein describe the first case of microcarcinoids with diffuse NE cell hyperplasia occurring in a patient with a history of congenital imperforate anus and colovesicular fistula, which resulted in prolonged exposure of colonic mucosa to urinary content.

## 2. Case Presentation

A 29-year-old male presented to our tertiary facility with imperforate anus and associated urinary incontinence. The patient had a history of multiple congenital abnormalities detected at birth, including atrial septal defect (ASD), persistent left-sided superior vena cava, cardiac conduction abnormalities, Wolff-Parkinson-White (WPW) syndrome, and vertebral anomalies. The gastrointestinal and genitourinary abnormalities were comprised of malformations including imperforate anus with the rectum terminating to the left and above the sphincteric complex, a connection between the rectum and bladder, and left vesicoureteral reflux.

He underwent open cardiac surgery for repair of the ASD and ablation of the WPW foci. Also, shortly after birth, the patient underwent surgery during which a loop transverse colostomy was created and an anoplasty was attempted unsuccessfully. Subsequently at the age of 26, a posterior sagittal anorectoplasty could not be completed due to technical difficulties. For the past several years, the patient had stool from the proximal portion of his colostomy and urine from the distal nonfunctional limb with persistent exposure of both colostomy limbs to urine and fecal material.

**Figure 1:**
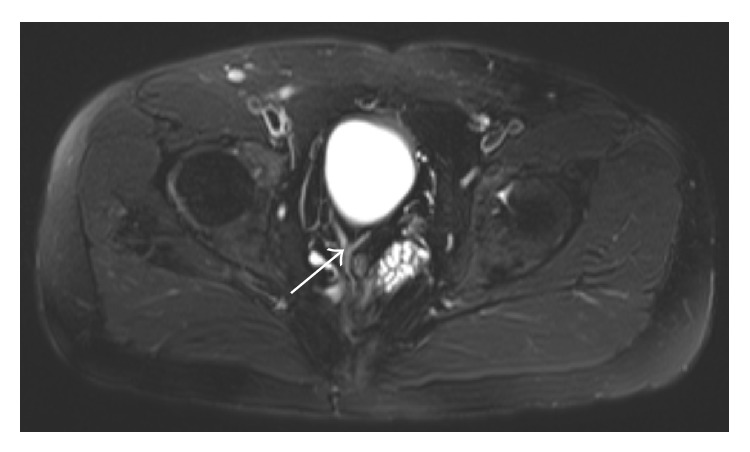
MRI of pelvis at the level of the bladder and rectum. Arrow: colovesicular fistula.

On physical examination, a left upper quadrant colostomy and imperforate anus were confirmed. MRI demonstrated the abnormalities stated above and suggested a mass near the colovesicular fistula (Figure 1). Endoscopic evaluation with colonoscopy and cystoscopy with biopsies did not demonstrate any pathologic alterations. Examination under anesthesia with muscle stimulation demonstrated functional anal sphincter muscles. Thus, the patient underwent takedown of the colovesicular fistula, closure of the bladder neck, reimplantation of the right ureter, small bowel resection with creation of an ileovesicostomy, colostomy takedown, colocolonic anastomosis, low anterior resection, posterior sagittal anorectoplasty, and coloanal anastomosis with a diverting loop ileostomy. He recovered well from the surgery without complications.

**Figure 2:**
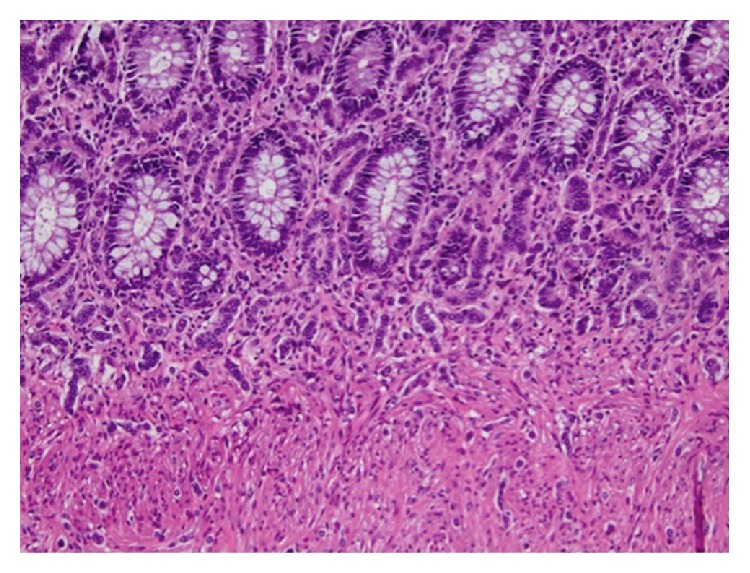
Clusters and cords of medium-sized cells with small round to oval nuclei dispersed within the colonic mucosa (H&E, ×200).

**Figure 3:**
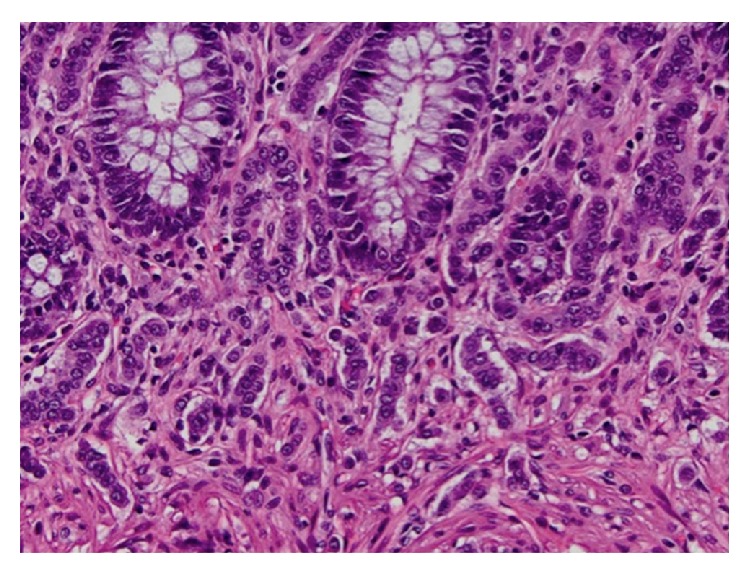
At high power, the cells contain fine chromatin. Nucleoli and mitoses are inconspicuous (H&E, ×400).

**Figure 4:**
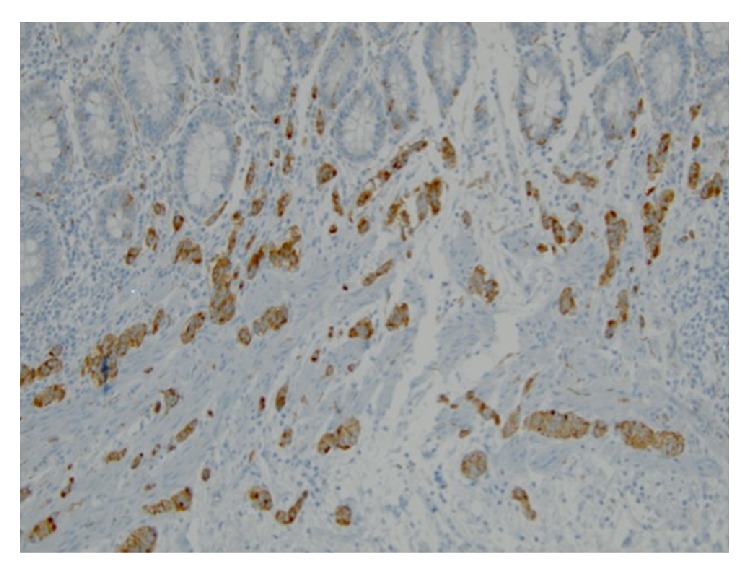
Strong and diffuse immunoreactivity with synaptophysin confirms neuroendocrine differentiation (Synaptophysin, ×200).

**Figure 5:**
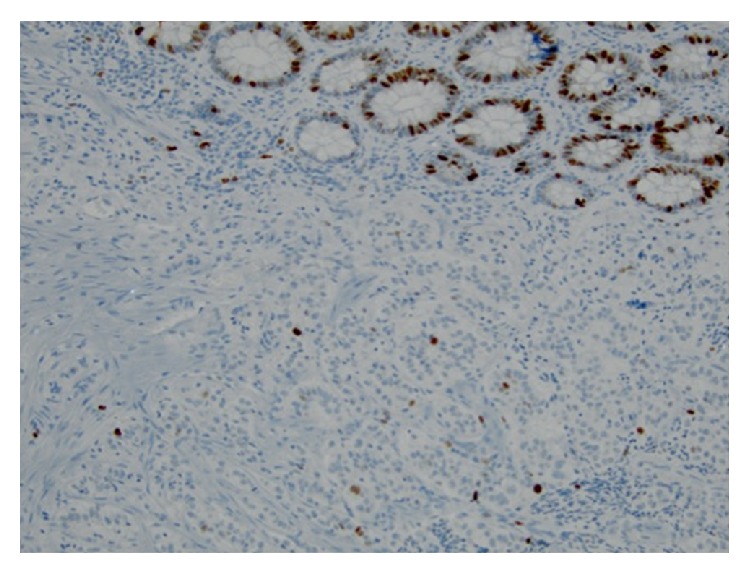
The proliferation index was <2% (Ki-67, ×200).

The specimen consisted of a segment of rectum containing the colovesicular fistula and a segment of sigmoid colon submitted separately, measuring 8.2 cm and 6.8 cm in length, respectively. Examination of initial representative sections revealed the incidental finding of neuroendocrine cell hyperplasia, after which the entire rectal specimen was submitted for microscopic evaluation. Histologic sections of the rectum and sigmoid demonstrated multiple microscopic foci of neuroendocrine cell hyperplasia with rare foci of microcarcinoids, the largest of which measured 5 mm in greatest dimension. These foci involved the mucosa and submucosa and were composed of small clusters of medium-sized cells with small round to oval nuclei containing fine chromatin with a salt and pepper appearance (Figures 2 and 3). The mitotic rate was 1 per 10 high power fields. Necrosis was absent. By immunohistochemistry, the lesional cells were strongly and diffusely immunoreactive with synaptophysin and chromogranin (Figure 4). The proliferative index, assessed with Ki-67, was less than 2% (Figure 5). Additionally, serum chromogranin A levels were found to be normal.

## 3. Discussion

Review of the literature demonstrates a risk of malignancy in gastrointestinal segments exposed to urine following surgical correction of urinary tract abnormalities [3–5, 7]. Late occurrence of colonic adenocarcinoma after ureterosigmoidostomy has been reported with an incidence of 5 to 40%, representing a significant increase compared to the age-matched general population [1, 8, 9]. Also, a higher risk of malignancy in bowel segments after cystoplasty or urinary conduit reconstruction has been documented [3, 10, 11]. The most commonly reported cancers include adenocarcinoma and urothelial cell carcinoma; however, rare cases of squamous cell carcinoma have been reported as well [1]. Typically, these tumors develop within a few years to decades after the surgical procedure and are located at the anastomotic site [12].

While the physiopathologic mechanism is not well known, it has been postulated that contact among urine, feces, urothelium, and colonic epithelium at the anastomotic site of ureterosigmoidostomies activates fecal carcinogens [8, 13]. Also, bacteriuria and production of N-nitrosamine have been considered responsible factors for tumorigenesis [11, 12].

Neuroendocrine cell hyperplasia (NEH) is a well-established entity in the lung and in the upper gastrointestinal tract, particularly stomach [6] and pancreas. However, there is no such well-defined entity in the colon. NE tumors in the colon represent approximately 1-2% of colorectal neoplasms. In the rectum, this type of tumor typically occurs in the 6th decade of life and has a low propensity for lymph node involvement and distant metastasis [14–16].

While the association of NEH with certain entities such as inflammatory bowel disease and diversion colitis is well documented [2, 17, 18], to the best of our knowledge, there is only one case report describing NEH in colonic tissue used for cystoplasty [2]. Kochevar reports a case of adenocarcinoid tumor arising in an ureteroileal conduit [7]. These studies describe the development of different types of cellular hyperplasia and malignancy in intestinal mucosa in contact with urine after ureterosigmoidostomy or cystoplasty exposing intestinal mucosa to urine with no or low fecal stream. Our case is the first to show microcarcinoids and NEH in colonic mucosa exposed to urinary content due to the type of conduit implemented. Interestingly, in our case NEH was observed not only at the fistula site but also distantly in the sigmoid, suggesting a more diffuse phenomenon. There was no significant inflammation despite long-term exposure of colonic mucosa to urinary content. The biologic mechanism of this finding is not known; however, the possibility of a process akin to that described in patients with ureterosigmoidostomy and cystoplasty who develop carcinoma has been raised.

## 4. Conclusion

Review of the literature demonstrates that few cases have been reported exhibiting the occurrence of different types of malignancy in association with long-term exposure of colonic mucosa with urinary content. Our case shows development of NEH and microcarcinoids in association with long-term exposure of colonic mucosa to urinary content. While uncommon, the reported cases establish the possibility of development of malignancy including NE neoplasms in the urointestinal tract postoperatively. Thus, one may recommend long-term follow-up of those patients who have undergone a surgical procedure exposing intestinal mucosa to urinary contents with or without fecal stream.
